# aEYE: A deep learning system for video nystagmus detection

**DOI:** 10.3389/fneur.2022.963968

**Published:** 2022-08-11

**Authors:** Narayani Wagle, John Morkos, Jingyan Liu, Henry Reith, Joseph Greenstein, Kirby Gong, Indranuj Gangan, Daniil Pakhomov, Sanchit Hira, Oleg V. Komogortsev, David E. Newman-Toker, Raimond Winslow, David S. Zee, Jorge Otero-Millan, Kemar E. Green

**Affiliations:** ^1^Department of Biomedical Engineering, The John Hopkins University, Baltimore, MD, United States; ^2^Department of Computer Science, The Johns Hopkins University, Baltimore, MD, United States; ^3^The John Hopkins University School of Medicine, Baltimore, MD, United States; ^4^Institute for Computational Medicine, The Johns Hopkins University, Baltimore, MD, United States; ^5^Department of Computer Science, Texas State University, San Marcos, TX, United States; ^6^Departments of Ophthalmology and Otolaryngology, The John Hopkins University School of Medicine, Baltimore, MD, United States; ^7^Department of Emergency Medicine, The John Hopkins University School of Medicine, Baltimore, MD, United States; ^8^Departments of Electrical and Computer Engineering, The John Hopkins University, Baltimore, MD, United States; ^9^Department of Neurosciences, The John Hopkins University School of Medicine, Baltimore, MD, United States; ^10^Department of Neurosciences, The John Hopkins University School of Medicine, Baltimore, MD, United States; ^11^School of Optometry University of California–Berkeley, Berkeley, CA, United States

**Keywords:** nystagmus, vertigo, artificial intelligence, dizziness, machine learning, telemedicine, deep learning, eye movements

## Abstract

**Background:**

Nystagmus identification and interpretation is challenging for non-experts who lack specific training in neuro-ophthalmology or neuro-otology. This challenge is magnified when the task is performed *via* telemedicine. Deep learning models have not been heavily studied in video-based eye movement detection.

**Methods:**

We developed, trained, and validated a deep-learning system (aEYE) to classify video recordings as normal or bearing at least two consecutive beats of nystagmus. The videos were retrospectively collected from a subset of the monocular (right eye) video-oculography (VOG) recording used in the Acute Video-oculography for Vertigo in Emergency Rooms for Rapid Triage (AVERT) clinical trial (#NCT02483429). Our model was derived from a preliminary dataset representing about 10% of the total AVERT videos (*n* = 435). The videos were trimmed into 10-sec clips sampled at 60 Hz with a resolution of 240 × 320 pixels. We then created 8 variations of the videos by altering the sampling rates (i.e., 30 Hz and 15 Hz) and image resolution (i.e., 60 × 80 pixels and 15 × 20 pixels). The dataset was labeled as “nystagmus” or “no nystagmus” by one expert provider. We then used a filtered image-based motion classification approach to develop aEYE. The model's performance at detecting nystagmus was calculated by using the area under the receiver-operating characteristic curve (AUROC), sensitivity, specificity, and accuracy.

**Results:**

An ensemble between the ResNet-soft voting and the VGG-hard voting models had the best performing metrics. The AUROC, sensitivity, specificity, and accuracy were 0.86, 88.4, 74.2, and 82.7%, respectively. Our validated folds had an average AUROC, sensitivity, specificity, and accuracy of 0.86, 80.3, 80.9, and 80.4%, respectively. Models created from the compressed videos decreased in accuracy as image sampling rate decreased from 60 Hz to 15 Hz. There was only minimal change in the accuracy of nystagmus detection when decreasing image resolution and keeping sampling rate constant.

**Conclusion:**

Deep learning is useful in detecting nystagmus in 60 Hz video recordings as well as videos with lower image resolutions and sampling rates, making it a potentially useful tool to aid future automated eye-movement enabled neurologic diagnosis.

## Introduction

**Figure 1:**
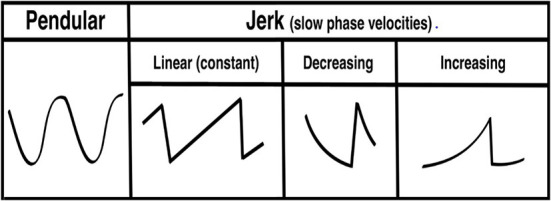
Pendular and jerk nystagmus waveform morphologies.

Nystagmus is an involuntary, rhythmic ocular instability that is initiated by an unwanted slow-phase drift in one direction followed by a corrective phase (fast or slow) in the opposite direction (1, 2). The waveforms can be divided into two morphologies: (1) pendular (sinusoid slow slow-phase drift and slow-phase correction) and (2) jerk (slow-phase drift and fast-phase correction). The “jerk” waveform can be further divided based on the velocity profile of the slow-phase into linear (or constant velocity slow-phase), velocity-decreasing and velocity-increasing (Figure 1). The pattern of nystagmus often localizes the underlying neural substrate that is damaged, and thus provides rapid diagnostic clues about changes in neurophysiology that occur in various lesions affecting these circuits. It has been shown that nystagmus and other subtle eye movement abnormalities are more sensitive and specific than early neuroimaging in distinguishing potentially devastating strokes from more benign inner ear problems (3, 4). These subtle findings are often missed by those on the frontlines in the emergency room and are often appreciated only by neurologists specializing in neuro-otology; there are < 50 of these providers practicing in the United States. Additionally, there are other “non-nystagmoid” movements such as square-wave jerks, ocular flutter, opsoclonus and other voluntary ocular oscillations that might pose a diagnostic challenge for both experts and non-experts alike.

The shift toward remote health assessment in the setting of the pandemic negatively impacted the quality of the physician's neurological assessment; the major limitation being the patient video capability. For nystagmus detection, simulated data of nystagmus waveforms showed that it is difficult to appreciate these subtle clinical findings on videos with lower frame rates (5). In the absence of an expert neuro-otologist/neuro-ophthalmologist, an automated model that could detect subtle degrees of nystagmus from video recordings, when video qualities poor, would be a boon to frontline practitioners facing a diagnostic challenge.

Others have successfully classified nystagmus from waveforms directly (6–9) and by generating waveforms from recorded videos (10, 11) using various machine/deep learning methods. Classification of nystagmus from video motion features independent of the calibration and calculation of eye movement velocity has only been rarely attempted (12). We developed, trained, and validated an artificial intelligence (AI)-based deep learning model (aEYE) directly from video recordings and investigated its utility as a potential screening tool for video nystagmus detection in various simulated video recording conditions.

## Methods

### Study design and dataset description

**Figure 2:**
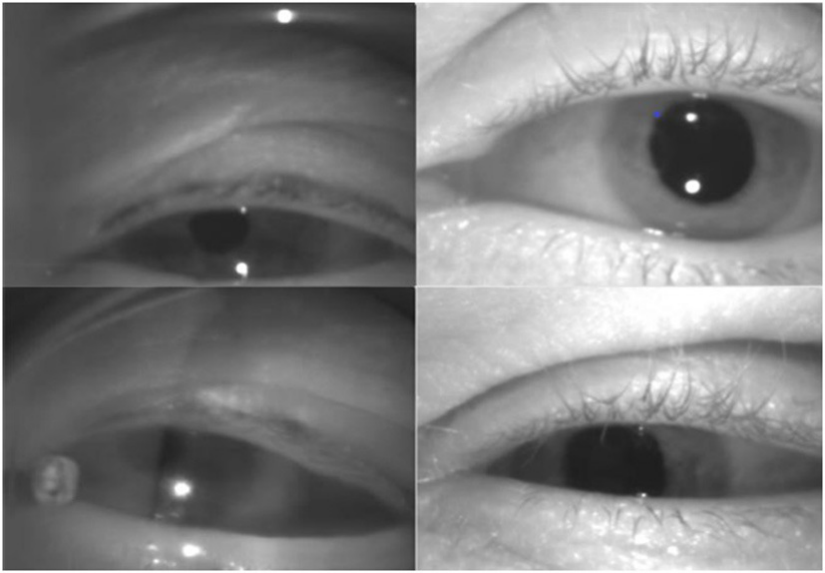
Variations in video quality of the dataset.

aEYE was developed using infrared monocular video-oculography recordings retrospectively obtained from the AVERT (13) research dataset. The AVERT trial is a multicenter, randomized clinical trial comparing the diagnostic accuracy of care guided by video oculography (VOG)-based eye movement recordings (supervised decision support) vs. standard care in diagnosing patients with acute dizziness and vertigo in the emergency department (ClinicalTrials.gov #NCT02483429). Both the AVERT trial and the current study were approved by the Johns Hopkins University School of Medicine's Institutional Review Board (IRB). Our dataset consisted of 435 monocular infrared VOG recordings of dizzy patients that represented about 10% of the total AVERT video dataset. All videos used were obtained from 30 patients. In the AVERT dataset, the videos were recorded in primary gaze, eccentric gaze, Dix-Hallpike, supine head roll, bow, lean and post-horizontal headshaking. Primary and eccentric gaze videos were recorded with and without visual fixation, while all others were recorded without visual fixation. In the videos that contained nystagmus (*n* = 218), 95% were linear jerk (vestibular nystagmus) and 5% velocity decreasing jerk (gaze-evoked nystagmus); there were no pendular nystagmus videos in the dataset. Of the 95% of linear jerk nystagmus videos, 9.1% (*n* = 20) had nystagmus in primary gaze; the remainder were from positional testing. All videos were of the right eye; nystagmus in dizziness is almost always the same in both eyes. All videos were recorded using the Natus/Otometrics ICS Impulse infrared VOG googles (14). Recordings were all grayscale and sampled at 60 Hz and had a resolution of 240 × 320 pixels. We then simulated 8 variations of videos of varying sampling rates (i.e., 30 Hz and 15 Hz) and image resolution (i.e., 60 × 80 pixels and 15 × 20 pixels). Only the first 10 sec of each video (600 frames) were used. The quality of the recording (i.e., lighting and visualization of the eyes) varied as shown in Figure 2. Each video was labeled as “nystagmus” or “no nystagmus” by one expert Neuro-otologist (K.E.G.) based on the presence of two consecutive slow and fast phase alternations (beats). The “nystagmus” to “no nystagmus” in our dataset was approximately 1:1; the train to test split was about 3:1. There was equal representation of videos from both classes from each of the 30 patients in both the training and test sets to account for potential bias in the model. The best performing model was then validated using k-fold cross-validation with k = 3 folds.

### Filtered image construction

We adopted and modified a previously described recursive filtering method (15, 16) used for detecting walking for the purposes of detecting ocular movements. A filtered image was created by applying recursive filtering to a 10-sec grayscale video clip (600 frames). This creates a representation of video motion based on the idea that a filtered image (***F***_***t***_) at time **(*t*)** is defined as the absolute value of the difference between a raw video frame (***I***_***t***_) at time **(*t*)** and an intermediate image (***M***_*t*_) at time **(*t*)** that combines content of raw video frames prior to time point ***t***.


$$\begin{array}{l} F_{t}=\left|I_{t}-M_{t}\right|\\ M_{t}=\left(1-\beta \right)M_{t-1}+\beta I_{t-1}\\ M_{0}=I_{1}\\ \text{where t=1,2,…,n} \end{array}$$

**Figure 3:**
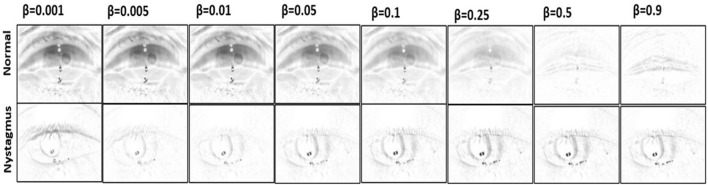
Filtered image appearance across different beta values.

The appearance of the filtered image can be modified by changing the parameter (**β)** that control the weights of the prior context of the intermediate image **(*M*)** and the raw video frame **(*I)*** as shown in Figure 3.

**Figure 4:**
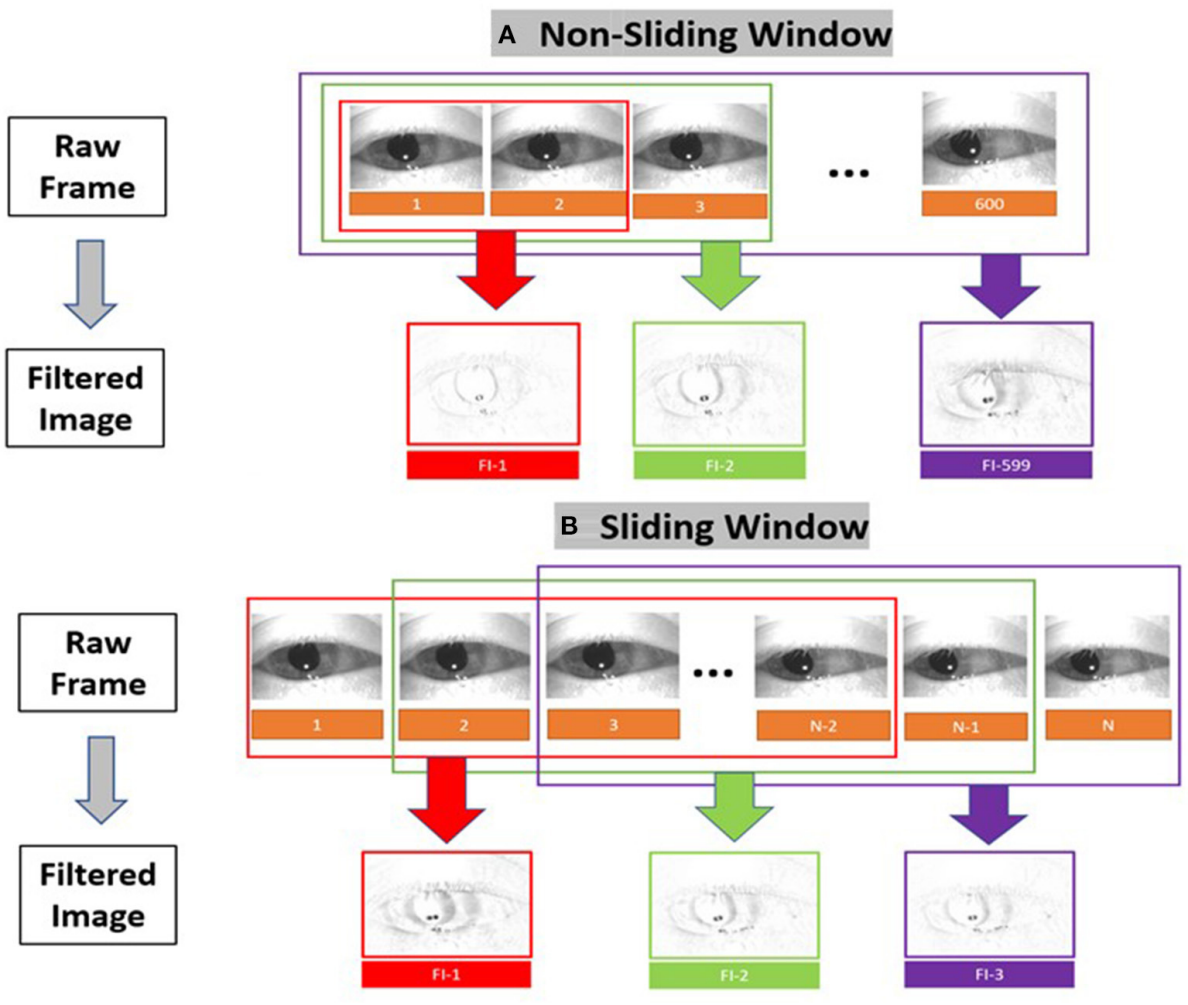
Filtered image construction for **(A)** the non-sliding window and **(B)** the sliding window variations.

The result is a set of images containing at maximum 599 filtered images that depicts motion at points in time as shown in Figure 4. In addition to the “original” filtered image (non-sliding window), we also constructed sliding window filtered images [10 frames (150 ms), 20 frames (333 ms), 30 frames(500 ms), and 60 frames(1s)] to consider the temporal criteria for slow and fast phase combinations (i.e., beats) of nystagmus.

### Video quality variations

**Figure 5:**
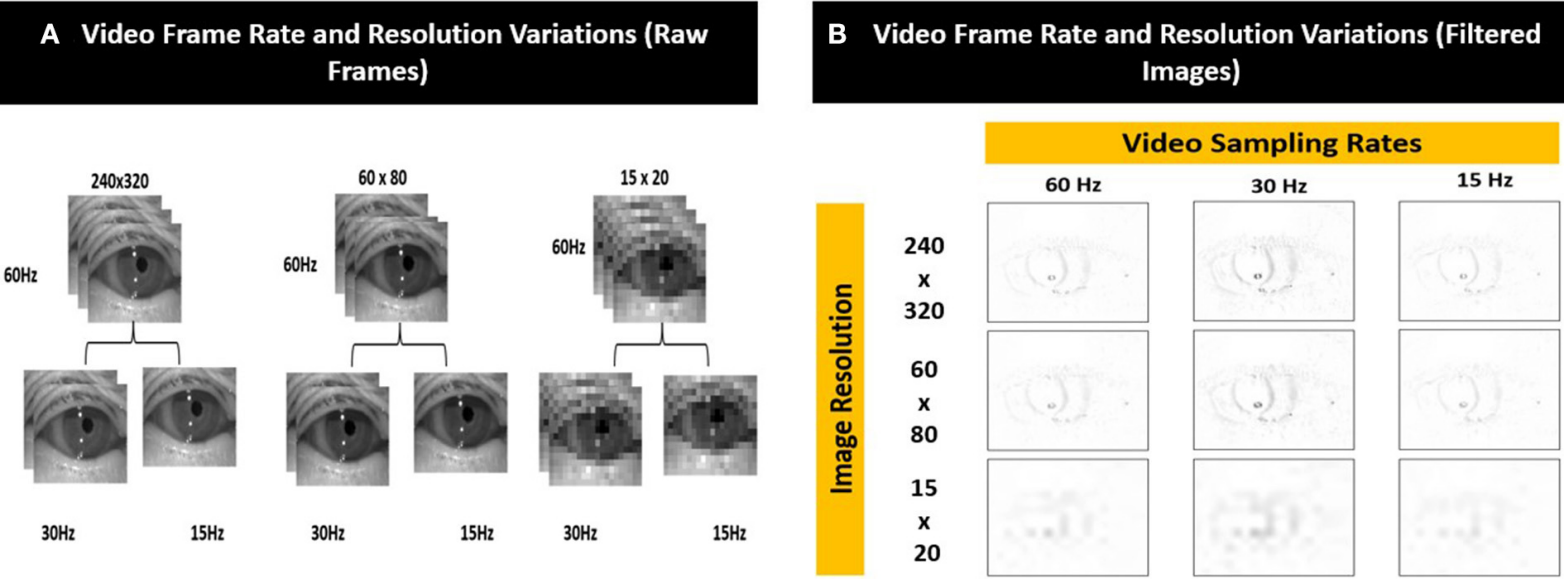
Examples of the different video frame rate and resolution variations for **(A)** raw frames and **(B)** corresponding filtered images.

We simulated 8 variations of videos from the original recordings (i.e., sampling rate = 60 Hz and resolution = 240 × 320 pixels) of varying sampling rates (i.e., 30 Hz and 15 Hz) and image resolution (i.e., 60 × 80 pixels and 15 × 20 pixels) as shown in Figure 5A. The compressed images were then converted into filtered images (Figure 5B) using the method illustrated in Figure 4.

### Deep learning model (aEYE) architecture

**Figure 6:**
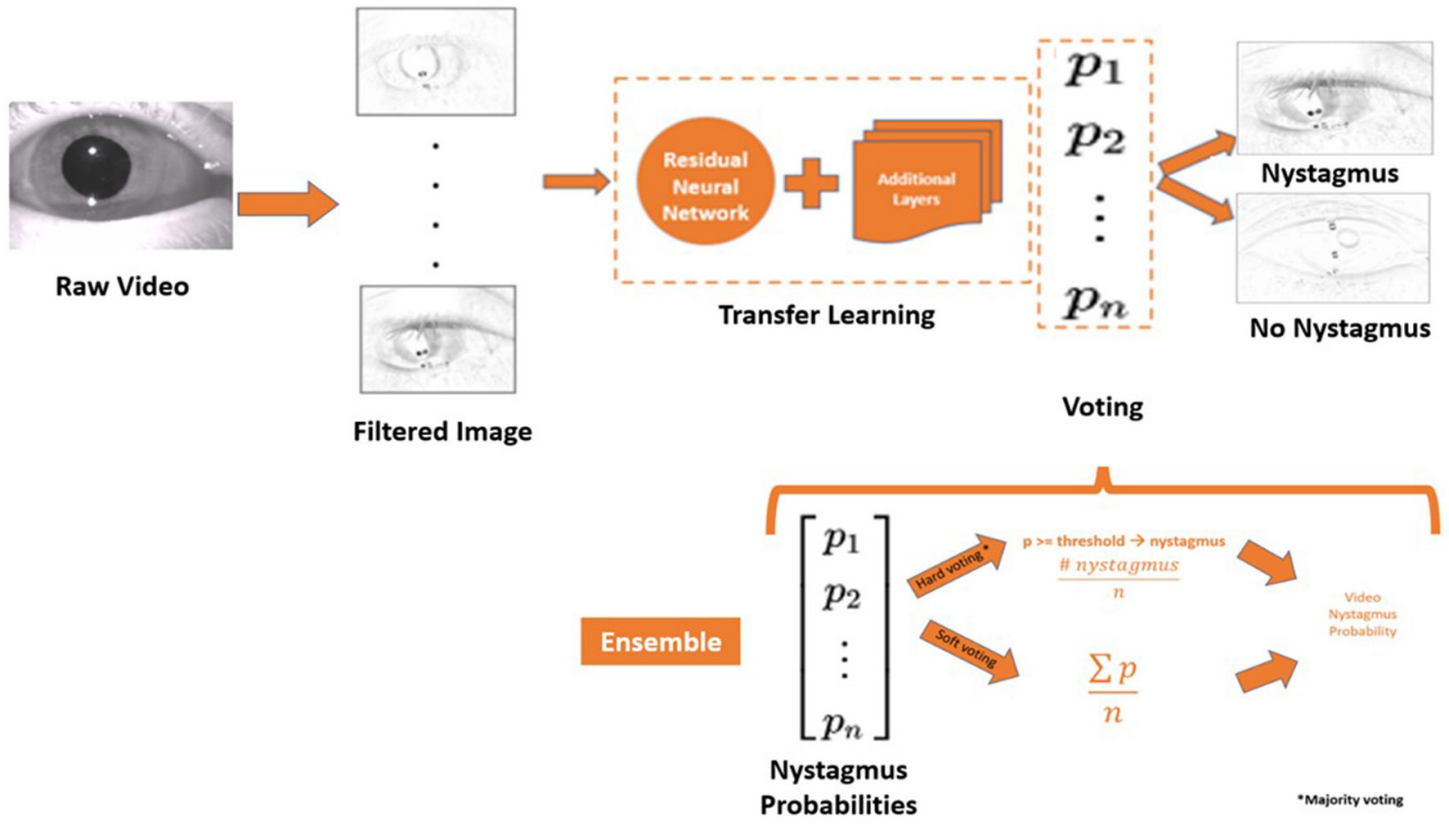
Model architecture and the framework of the ensemble model. *P*, probability; *n*, number of filtered images; ∑, the sum of; *n*, number of filtered images.

The proposed motion classification algorithm is a filtered image-based (16) approach (Figure 6). The filtered image-based motion classification algorithm uses a set of filtered images as video motion data. Each filtered image is labeled according to the label from the video it was generated and used to train a classifier in a supervised fashion. We trained classifiers from the ImageNet dataset (ResNet) using transfer learning approaches to detect nystagmus from filtered images. The videos in each test set were equally balanced between “nystagmus” and “no nystagmus”. aEYE was trained and tested to yield a binary class prediction of “nystagmus” or “no nystagmus” for each filtered image in the test set. Hard (majority) voting was performed to summate the filtered images from each video that were classified as nystagmus. In hard voting, only videos that have an average probability of nystagmus amongst all filtered images (*n* = 599) are classified as nystagmus (17). We also experimented with a form of soft voting where every individual filtered image provides a probability value that a specific video belongs to a particular target class (nystagmus or no nystagmus).

### Voting based on the temporal criteria of nystagmus

Nystagmus as defined in our dataset is at least two contiguous cycles (beats) of alternating fast and slow phase combinations along any plane. The duration of a slow phase can vary from ~150 msec (~10 frames) to ~350 msec (~20 frames)—closely representing the duration of a beat. The aim of this experiment is to determine if aEYE's performance can be improved by changing the voting method to identify consecutive filtered images bearing a probability of nystagmus. We experimented with the following four temporal voting criteria: (1) ≥50 consecutive frames (~750 ms), ≥100 consecutive frames (~1,500 ms); ≥150 consecutive frames (~2,250 ms), and ≥350 consecutive frames (~5,250 ms).

### Data split modifications

Based on the filtered image misclassifications (false positives and false negatives) videos where the eyes were eccentrically positioned (i.e., not looking straight ahead) ranked the highest. We evaluated how an equal split of eccentrically positioned videos in both the training and test sets impacted the model's performance.

### ImageNet classifier comparison

Classifiers for the ImageNet dataset, such as ResNet, DenseNet, VGG and Inception which perform better on medical data, were trained to detect nystagmus from filtered images (16). Nystagmus detection was compared across all four ImageNet classifiers.

### Ensemble model

Ensemble techniques involve model averaging that is aimed at reducing generalization errors. We applied an ensemble technique like bootstrap aggregating (or bagging) (19) where different models are trained separately (on the same dataset), and the output is determined by averaging different voting methods. Hard and soft voting methods were used to create an ensemble model (Figure 5) that averages both voting methods to decide on the final class designation (nystagmus or no nystagmus).

### Comparison with existing video classification method

We adopted a simple long short-term memory (LSTM) and convolutional neural network (CNN) model (18) without any frame sampling that has been shown to produce state-of-the-art performance for other action recognition problems.

### Statistical analysis

The performance was based on the model's detection of videos with or without nystagmus. The performance of the models was calculated using the area under the receiver operating characteristic curve (AUROC) at the operating point with accuracy sensitivity and specificity. It is important to note the differences between accuracy (the ratio between the number of correctly predicted samples to the total number of samples) and AUROC (the ratio of the false positive and true positive rates at different probability thresholds of the model's prediction) (19). We also compared the best AUROC for each model experiment to the best overall AUROC using two sample (unpaired) *t*-test. A *p*-value < 0.05 indicates evidence of statistical difference between two experiments.

**Figure 7:**
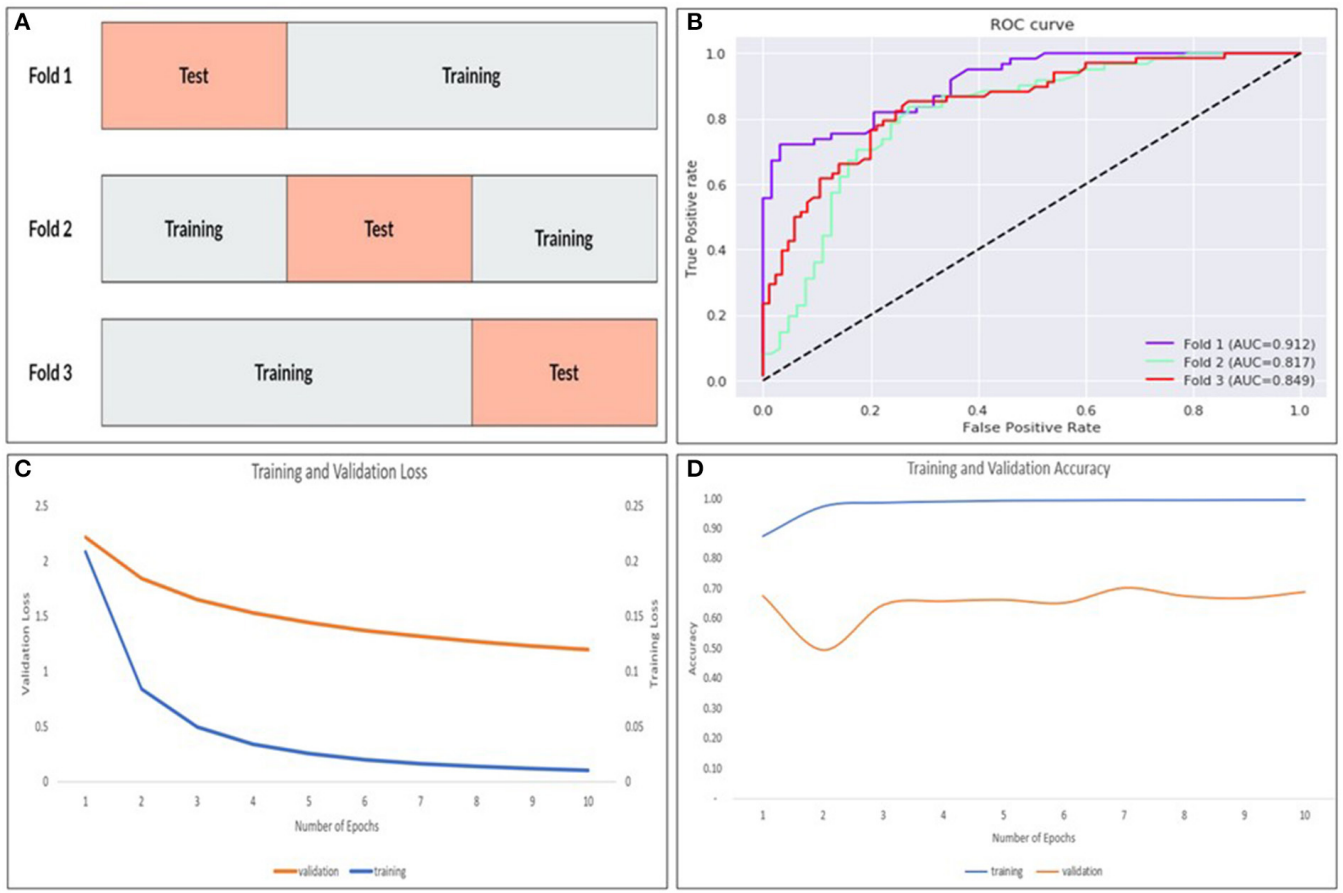
The k-fold cross validation architecture with the data partitioned into training and tests sets for k = 3–folds **(A)**, AUROC curves for each fold **(B)** as well as training/validation loss **(C)** and training/validation accuracy **(D)** for the model.

Internal validation of our best performing model was done using stratified k-fold cross-validation (19) that partitions our data into k = 3 folds as demonstrated in Figure 7A. Each fold contains an evenly distributed sample of both class. In the validation experiments, we also ensured that there was equal representation of videos in both classes from an individual patient. To maintain these constraints, it was not feasible to balance video heterogeneity. The performance was measured in aggregate across each test set. Each subset was stratified, ensuring an even class split in each set.

## Results

### Filtered image optimization

The filtered image calculation described above includes a free parameter (**β*)*** that controls their temporal dynamics. We tested the performance of the best performing ResNet model (non-sliding window and soft voting*)* with 7 different **β** values (0.001, 0.005, 0.01, 0.05, 0.1, 0.25, and 0.5—see Figure 2 and Table 1). Filtered images obtained with **β** of 0.25 resulted in the highest accuracy (79.3%) at detecting nystagmus; however, specificity was low (69.3%)—implying a high false positive rate.

**Table 1:** Performance metrics for model experiments.

	**AUROC**	**Sensitivity**	**Specificity**	**Accuracy**
**Filtered image optimization**				
β = 0.001	0.75	68.1%	77.4%	72.5%
β = 0.005	0.79	81.1%	62.9%	72.5%
β = 0.01	0.83	84.0%	69.3%	77.1%
β = 0.05	0.78	60.8%	80.6%	70.2%
β = 0.1	0.81	71.0%	80.6%	75.5%
β = 0.25	0.85	88.4%	69.3%	79.3%
β = 0.5	0.79	75.3%	74.1%	74.8%
**Sliding window comparison**				
150 msec	0.80	78.3%	75.8%	77.1%
333 msec	0.80	75.4%	77.4%	76.3%
500 msec	0.82	71.0%	83.9%	77.1%
1,000 msec	0.81	72.5%	82.3%	77.1%
2,000 msec	0.75	61.0%	75.8%	67.9%
**Voting (*****β*** **=** **0.25)**				
Hard voting	0.84	72.4%	83.8%	77.8%
Soft voting	0.85	88.4%	69.3%	79.3%
**Sliding window-temporal voting criteria**				
1,000 msec−50 frames	0.77	60.9%	88.7%	74.1%
500 msec−50 frames	0.78	75.4%	74.2%	74.8%
333 msec−50 frames	0.74	71.0%	71.0%	71.0%
150 msec−50 frames	0.74	71.0%	71.0%	71.0%
1,000 msec−100 frames	0.70	47.8%	90.3%	67.9%
500 msec−100 frames	0.71	52.2%	88.7%	69.5%
333 msec−100 frames	0.69	49.3%	87.1%	67.2%
150 msec−100 frames	0.71	59.4%	82.3%	70.2%
1,000 msec−150 frames	0.67	36.2%	96.8%	64.9%
500 msec−150 frames	0.68	46.4%	88.7%	66.4%
333 msec−150 frames	0.67	44.9%	87.1%	64.9%
150 msec−150 frames	0.69	49.3%	87.1%	67.2%
1,000 msec−350 frames	0.59	20.3%	96.8%	56.5%
500 msec−350 frames	0.62	27.5%	95.2%	59.5%
333 msec−350 frames	0.58	17.4%	98.4%	55.7%
150 msec−350 frames	0.61	27.5%	93.5%	58.8%
**Data split modification**				
Balanced eccentric gaze videos	0.83	83.1%	72.1%	77.8%
**ImageNet classifier comparison**				
ResNet	0.84	72.4%	83.8%	77.8%
DenseNet	0.81	75.3%	79.0%	77.1%
VGG	0.85	59.4%	96.7%	77.1%
Inception	0.82	84.0%	72.5%	78.6%
**Ensemble**				
ResNet-soft vote	0.85	88.4%	69.3%	79.3%
VGG-hard vote	0.85	59.4%	96.7%	77.1%
ResNet-soft vote + VGG-hard vote ensemble*	0.86	88.4%	74.2%	81.7%
**ResNet-soft vote** **+** **VGG-hard vote ensemble model in reverse**				
Reverse model	0.50	0.00%	100%	47.7%
**Stratified k-fold cross validation (k** **=** **3–folds)**				
Fold 1	0.91	72.1%	97.0%	84.7%
Fold 2	0.82	83.6%	73.0%	78.3%
Fold 3	0.85	85.3%	72.9%	78.4%
Average	0.86	80.3%	80.9%	80.4%
**Comparison with existing video classification method**				
LSTM + CNN	0.46	100%	2.00%	48.4%
**Frame rate/resolution combinations**				
60 Hz (240 ×320)	0.86	88.4%	74.2%	81.7%
60 Hz (60 ×80)	0.84	78.3%	83.9%	80.9%
60 Hz (15 ×20)	0.83	68.1%	85.5%	76.3%
30 Hz (240 ×320)	0.83	76.8%	77.4%	77.1%
30 Hz (60 ×80)	0.85	71.0%	87.1%	78.6%
30 Hz (15 ×20)	0.83	89.9%	66.1%	78.6%
15 Hz (240 ×320)	0.81	65.2%	83.9%	74.1%
15 Hz (60 ×80)	0.82	78.3%	74.2%	76.3%
15 Hz (15 ×20)	0.72	55.1%	82.3%	67.9%

AUROC, area under the receiver operating characteristic curve; CNN, convolutional neural network; LTSM, long-term short-term memory. (*)indicating the best performing model.

### Sliding windows comparison

Filtered images with a beta value of 0.25 were used to carry out the sliding window experiments. The filtered images were created based on the following sliding windows: 10 frames (150 ms), 20 frames (333 ms), 30 frames (500 ms), and 60 frames (1 s). None showed any improvement in the model's overall performance.

### Voting

Our neural network classifies individual filtered images as containing or not containing nystagmus. However, to evaluate the results against our labeled data, we classified the entire video. We then compared different voting strategies where each image votes toward the result of the video classification. Hard (majority voting) and soft voting techniques were compared using the value of **β** previously determined to be best (**β** = 0.25). While the soft voting model had a slightly higher AUROC (0.85), there was better overall sensitivity (72.4%) and specificity (83.8%) with majority voting (Table 1).

### Sliding window-temporal voting criteria

The result of combining the sliding window models with different temporal voting criteria is shown in Table 1. As we increased the number of consecutive frames, the overall AUROC and sensitivity decreased while the specificity increased.

### Data split modification

In our dataset (*n* = 435 videos), 44.3% (*n* = 193) of the videos had eccentric gaze positioning of the eye during the recording. As shown in Table 1, there were no improvement in any of the measured performance parameters in the model accounting for equal eccentric gaze splits.

### ImageNet classifier comparison

We compared our best performing ResNet trained model (β = 0.25, non-sliding window and soft voting) to models trained on different ImageNet dataset (DenseNet, VGG and Inception) with the same β, sliding window and voting parameters. As shown in Table 1, the VGG model had a slightly higher AUROC (0.85); however overall sensitivity and specificity were better with the ResNet and Inception models.

### Ensemble model

Bootstrap aggregating techniques (17) were applied to the best performing models from the ImageNet classifier comparison experiments with different hard and soft voting combinations. The ResNet-soft vote + VGG-hard vote ensemble model had the most ideal performance metrics (Sensitivity = 88.4%; Specificity = 74.1%) of all the models as shown in Table 1. When the same model was tested with reversed videos, it performed poorly (AUROC = 0.50).

### Stratified k-fold cross validation

The ResNet-soft vote + VGG-hard vote ensemble model (best performing model) was cross validated using stratified k-fold cross validation. As shown in Figure 7B and Table 1, fold 1 returned the highest accuracy (84.7%), and there was a mean accuracy of (80.4%) across all 3–folds. Learning curves (Figures 7C,D) showed that our training loss follows a consistent trend and begins to converge early in training—indicating no overfitting.

### Comparison with existing video classification method

As expected, the LSTM + CNN model performed poorly (AUROC = 0.46) compared to (AUROC = 0.86) in the best performing filtered image model as shown in Table 1.

### Frame rate/resolution combinations

As shown in Table 1, the best performing model (AUROC = 0.86) was obtained from the videos with the original image specifications (i.e., sampling rate = 60 Hz and resolution = 240 × 320 pixels). Overall, there was a decrease in accuracy as image sampling rate decreased from 60 Hz to 15 Hz; however, there was only minimal change in the accuracy of nystagmus detection when image resolution was decreased while sampling rate was kept constant.

### AUROC comparison

As shown in Table 1, the overall best performing model (ResNet-soft vote + VGG-hard vote ensemble) had an AUROC of 0.86. Of the *n* = 131 predictions from the test samples, the mean model prediction probability was 0.251 with a standard deviation (SD) of 0.159. As shown in Table 2, no statistical difference (*p* = 0.837) exists between the AUROC of the overall best performing model and the 500 msec-50 frames model from the sliding window-temporal voting criteria experiments. There were statistically significant differences (*p* ≤ 0.05) between the AUROC of the ResNet-soft vote + VGG-hard vote ensemble model and the best AUROC for all the remaining experiments.

**Table 2:** Comparing best overall AUROC (ResNet-soft vote + VGG-hard vote ensemble) with best AUROC from each model experiment shown in Table 1 using two sample (unpaired) *t*-test.

**Experiment**	**Best AUROC**	**Mean MP**	**SD**	***p*-value**
**AUROC Comparisons**				
Filtered image optimization	0.85	0.529	0.369	<0.001
Sliding window comparison	0.82	0.518	0.379	<0.001
Voting (β = 0.25)	0.85	0.599	0.340	<0.001
Sliding window-temporal voting criteria	0.78	0.244	0.356	0.837
Data split modification	0.83	0.558	0.369	<0.001
ImageNet classifier comparison	0.85	0.439	0.328	<0.001
ResNet-soft vote + VGG-hard vote ensemble in reverse	0.50	0.000	0.000	<0.001
Comparison with existing video classification method	0.46	0.492	0.008	<0.001
Frame rate/resolution combinations (15 Hz)	0.82	0.604	0.292	<0.001
Frame rate/resolution combinations (30 Hz)	0.85	0.517	0.331	<0.001

*AUROC, area under the receiver operating characteristic curve. MP, Model Predictions; SD, Standard Deviation*.

## Discussion

We developed aEYE (a new deep learning method for nystagmus detection) from videos using non-traditional eye tracking techniques. Traditional nystagmus detection involves tracking the change in eye position over time using the pupil or other ocular features (e.g., the iris) (1, 20), which serves as surrogates for eye movements. With these methods, you can appreciate the quick and slow phases that define nystagmus—allowing for detection from characteristic nystagmus waveform morphologies. For the most robust nystagmus waveform detection, high frame rate video recordings are necessary for extraction of the precise ocular position data. The use of mobile devices (especially during the pandemic) has shifted the focus of eye tracking toward mobile solutions. While current mobile VOG technology exists (21), we are still not clear on their accuracy in detecting nystagmus since most mobile devices have relatively lower video qualities compared to standard eye trackers. Furthermore, simulated data of nystagmus waveform at different video sampling rates demonstrated degradation in the nystagmus waveform morphology after ~ 30Hz (5); other recording conditions such as recording distance may also play a role.

The result from our experiments suggests that the filtered image approach (15, 16) is well–suited for nystagmus detection from a relatively smaller dataset of low-quality videos. Reassuringly, the reproducibility of the model's performance in the stratified k-fold cross validation experiments suggests potential generalizability on external video datasets of similar size and quality. It is important to note that the video heterogeneity in our dataset (not accounted for in the validation experiments) implies that the model would likely perform better with less noise given fold 1's results (Table 1). In evaluating the performance of our model, the significance of the AUROC vs. the accuracy should be noted. For the AVERT patient population (all dizzy patients), the 1:1 split between nystagmus to “no nystagmus” is equivalent to what one would expect in that population, therefore the accuracy (81.7%) is a reliable measure of aEYE's performance; however, the AUROC (0.864) suggests a high probability of the model reproducing similar accuracies in “AVERT-like” patient population.

In the world of deep learning and image recognition, large datasets are often needed to ensure more accurate and reliable results (22, 23). In our study, we used a smaller video dataset (*n* = 435); however, since our input data was individual filtered images rather than the entire video clip, aEYE was trained on 179,700 (300 videos × 599 filtered images) data points. We believe this increased our model's performance tremendously, and probably explained why the non-sliding window model outperformed the sliding window models that created filtered images based on the temporal definition of a slow phase and contained fewer overall frames (see Table 1).

There are existing video classification methods (24) that uses forms of frame sampling for model input. Therefore, only a subset of the video frames is selected. Two consecutive beats of nystagmus can have a very short duration (as short as ~500 msec or ~30 frames) and is likely to be found in tiny chunks of the videos in our dataset (given the relative frequency of noise imparted by eye closure, blinks and other technical issues affecting video recording quality). As a result, implementing these methods risks eliminating the portions of our video that correspond to the class label (i.e., 2 consecutive beats of nystagmus). To counteract this, we used a simple LSTM and CNN model without any frame sampling (18). As suspected, the LSTM model performed poorly (AUROC = 0.46) as shown in Table 1. We believe the results seen may be due to one or both of the following factors. Since 600 frames per video was inputted, the complexity of the LSTM + CNN network was limited to handle the computational load. Additionally, video classification methods perform predictions at a video level while our proposed method performs predictions at an image level. With video classification, our dataset for training was ~ 300 videos whereas with our method, our dataset for training was ~ 180,000 filtered images.

**Figure 8:**
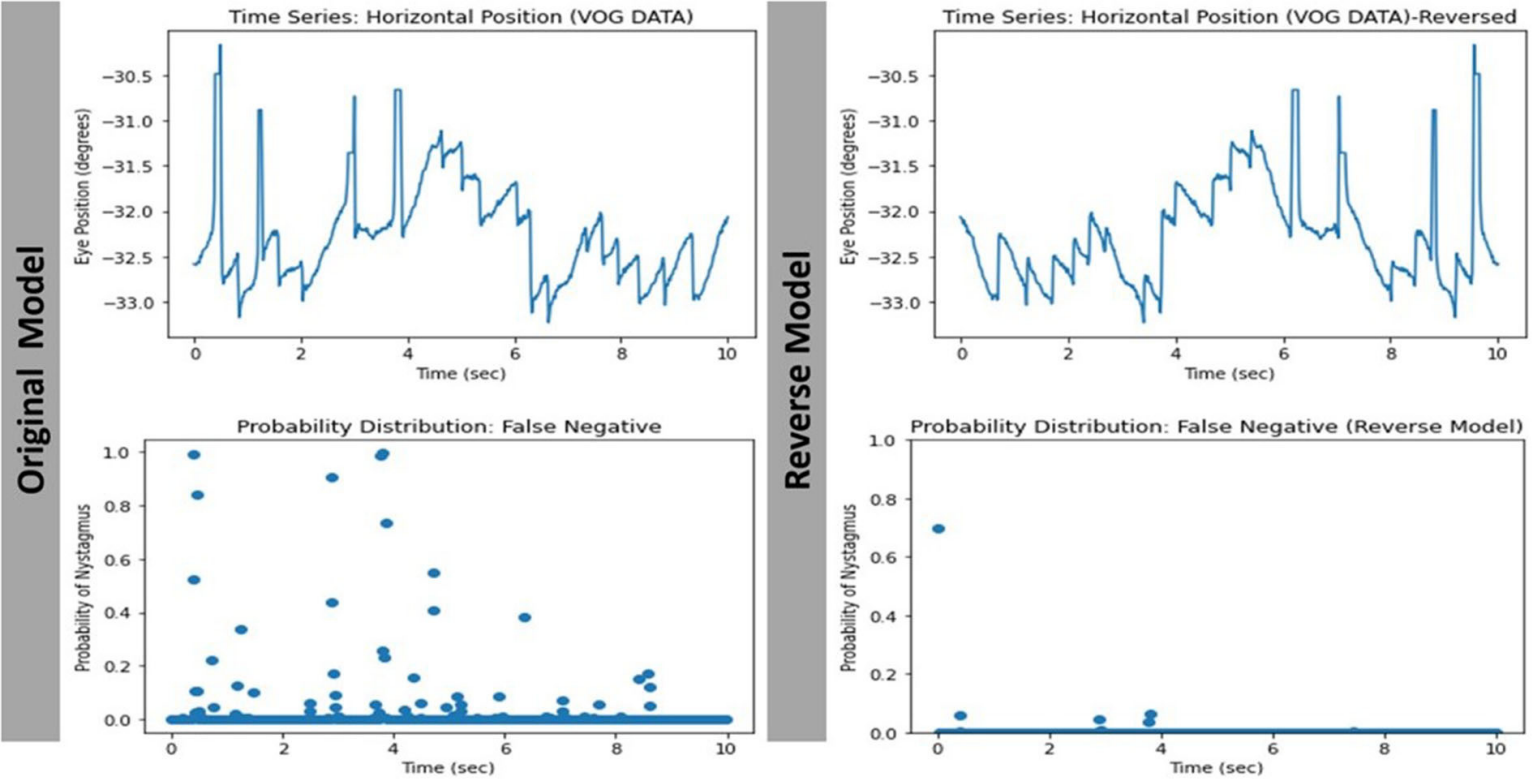
Comparing the filtered-image probability distribution of the same false negative video with nystagmus in eccentric eye position toward the end of video.

The way the model makes its prediction remains a mystery. We attempted to decipher this problem by studying the characteristics of the misclassified videos. Our evaluation revealed that 8/11 (72.7%) and 7/10 (70%) of the false positive and false negative cases respectively were videos with eccentric eye positioning (i.e., not looking straight ahead). Additionally, while analyzing learning curves (Figures 7C,D), unusual behavior was observed in our validation loss curve in folds 2 and 3. We speculate that this is driven by the fact that our validation sets only represent a small proportion of training data. Therefore, understanding misclassified videos may provide insight to improve model learning and optimization in training. We then hypothesized that we may be able to improve the model's performance if we had an equal split of eccentrically positioned videos in both the training and test sets. As shown in Table 1 there was no improvement in any of the performance parameters measured in the new balanced split model. One possible explanation for these findings was that most of the eccentrically positioned videos only had nystagmus at the end of the video. Since each filtered image carries motion information from earlier frames, the last filtered image will contain the motion information of the entire video. In our dataset, the 10 sec recordings may contain a plethora of non-nystagmus eye motion features (e.g., blinks, eye closure, square-wave jerks, etc,). Therefore, as we move from filtered image 1 to 599 in a nystagmus video with other motion features, it is possible that the average number of images with nystagmus probability will be much lower in the second half of the video. To test the second hypothesis, we created a reversed version of the non-sliding window model to decrease both the false positive and false negative rates. As shown in Table 1, the overall model performance was significantly worse. A closer look at the filtered image probability distributions of a false negative video example (containing nystagmus in eccentric eye position toward the end of video) in both the original and reverse model revealed an over lower number of filtered images with nystagmus probabilities in the reverse model (Figure 8).

The lack of a clear clinically relevant explanation for aEYE's prediction brings into question the likelihood that there might be other “markers” of nystagmus recognizable by the machine, that may not yet be apparent to clinicians. Future studies to investigate our deep learning model's decision making using novel and existing explainable AI methods will be needed to better understand predictions and decipher the “Blackbox” (25, 26).

## Conclusion

aEYE could be used for remote detection of nystagmus with potential future application in the detection of other eye movement types (square-wave jerks, ocular flutter, opsoclonus, etc). Further research into understanding the model's predictions using explainable AI (27) may be useful for improving the model's performance. Robust, international multicenter external cross validation will be needed to prove generalizability in different populations with various video recording capabilities.

## Data availability statement

The datasets presented in this article are not readily available because of ethical and privacy restrictions. Requests to access the datasets should be directed to KG, kgreen66@jhmi.ed.

## Ethics statement

The studies involving human participants were reviewed and approved by Johns Hopkins Medicine IBB. The patients/participants provided their written informed consent to participate in this study.

## Author contributions

NW, JM, JL, HR, JG, KG, SH, OK, RW, and DZ: data analysis and drafting and revision of manuscript. DN-T: drafting and revision of manuscript. JO-M: study concept and design, data analysis, and drafting and revision of manuscript. KG: study concept and design, data analysis, and drafting and revision of manuscript. All authors contributed to the article and approved the submitted version.

## Conflict of interest

Author DN-T conducts research related to diagnosis of dizziness and stroke, as well as diagnostic error. He serves as the principal investigator for multiple grants and contracts on these topics, including the NIH-sponsored AVERT clinical trial (NIDCD U01 DC013778, ClinicalTrials.gov #NCT02483429). Johns Hopkins has been loaned research equipment [video-oculography (VOG) systems] by two companies for use in DN-T's research; one of these companies has also provided funding for research on diagnostic algorithm development related to dizziness, inner ear diseases, and stroke. DN-T has no other financial interest in these or any other companies. DN-T is an inventor on a provisional patent (US No. 62/883,373) for smartphone-based stroke diagnosis in patients with dizziness. He gives frequent academic lectures on these topics and occasionally serves as a medico-legal consultant for both plaintiff and defense in cases related to dizziness, stroke, and diagnostic error.

The remaining authors declare that the research was conducted in the absence of any commercial or financial relationships that could be construed as a potential conflict of interest.

## Publisher's note

All claims expressed in this article are solely those of the authors and do not necessarily represent those of their affiliated organizations, or those of the publisher, the editors and the reviewers. Any product that may be evaluated in this article, or claim that may be made by its manufacturer, is not guaranteed or endorsed by the publisher.
